# Address of Dr. Willard

**Published:** 1858-04

**Authors:** 


*Address of Dr. Willard.*—We are happy to learn that the address of our estimable fellow-citizen, Dr. Augustus Willard, before the State Medical Society recently in session at Albany, and over which he had the honor to preside, was, in all respects, an able and scientific document, highly suggestive in its character, and worthy of the habitually close observation and sound deductions of the author. Those who knew Dr. Willard well, anticipated, of course, that his official address would challenge and deserve the attention of the learned professional body before whom it was delivered, and we are happy to learn, as we have hitherto remarked, that this expectation was fully realized.

Rural districts are not, for obvious reasons, congenial to a rapid growth of professional character. Cities afford a better field for the development and exhibition of skill in medicine and surgery, and for the more speedy realization of that measure of fame for which every professional man earnestly struggles; but yet the personal history of the medical profession proves, that the most profound physicians of the commonwealth have been found among those who have commenced and terminated their practice in the rural districts. Removed from scenes of excitement and pleasure, with an abundance of leisure to study and observe, to read and reflect, they become, if blessed with sound and discriminating intellects and industrious habits, the embodiments of professional wisdom and science, to whom may be committed, without hesitation, the treatment of the most delicate cases of disease. We have in our mind's eye a large number of this class, who have shown themselves public benefactors.

Among them we recognize Dr. Willard. He commenced the practice of his profession in this village, and has grown gray among us. Proverbially estimable in all the relations of life, he especially deserves the gratitude of this community for his professional services. Unobtrusive and unambitious, except in the discharge of his public and professional duties, the knowledge of his worth and merit is nevertheless co-extensive with the state, as the fact of his election as President of the State Medical Society—a body of learned and scientific gentlemen—abundantly proves. To those who have availed themselves of his ministrations in the sad hours of sickness, (and who, in this community, has not?) it is a pleasure to feel that so flattering a compliment was bestowed on him, the obligations of which he has so satisfactorily and so creditably discharged.

The mission of the physician is one of peculiar and delicate responsibility, the assumption of which can only be properly hazarded by one combining all the requisites of fine intellect, sound judgment, professional learning and skill, integrity and honor. Without these, the physician is a dangerous man anywhere. The delicate mechanism of the human system can not be trifled with, with impunity; the no less delicate trusts confided to him, when invited to enter the family circle or the sick-room professionally, can not be betrayed without dishonor; and it would be well if these young aspirants for professional fame would ponder these suggestions, and aim to find an exemplar in such a man as Dr. Willard.—*Chenango American.*

